# Full Pulpotomy as a Treatment for Irreversible Pulpitis in Permanent Teeth: A Systematic Review of the Literature Based on Case Reports

**DOI:** 10.7759/cureus.46808

**Published:** 2023-10-10

**Authors:** Vicente Rueda-Ibarra, Norma L Robles-Bermeo, Blanca S González-López, Carlo E Medina-Solís, José G Serrano-Robles, Sonia Márquez Rodríguez, Josué R Bermeo-Escalona, Victor J Delgado-Pérez, Gerardo Maupomé

**Affiliations:** 1 School of Dentistry, Autonomous University of the State of Mexico, Toluca, MEX; 2 Academic Area of Dentistry, Health Sciences Institute, Autonomous University of the State of Hidalgo, Pachuca, MEX; 3 Advanced Studies and Research Center in Dentistry "Dr. Keisaburo Miyata", School of Dentistry, Autonomous University of the State of Mexico, Toluca, MEX; 4 Center for Research in Health Sciences, Faculty of Health Sciences, Anahuac University North Campus, Ciudad de Mexico, MEX; 5 Department of Epidemiology, Richard M. Fairbanks School of Public Health, Indiana University, Indianapolis, USA

**Keywords:** vital pulp therapy, permanent teeth, irreversible pulpitis, full pulpotomy, oral health

## Abstract

The objective of this systematic review was to evaluate the current evidence of case reports where the treatment for permanent teeth with a diagnosis of irreversible pulpitis was a full pulpotomy. This study was carried out by two reviewers following the Preferred Reporting Items for Systematic Reviews and Meta-analyses (PRISMA) guidelines. A systematic electronic search was carried out in the PubMed, Google Scholar, and Scopus databases until the year 2022 to find articles in English where the treatment for irreversible pulpitis in permanent teeth was a full pulpotomy. Literature reviews, in vitro or animal studies, abstracts, and unpublished data were excluded. The intervention, control, and outcome parameters were selected following the “Population, Interventions, Control, and Outcome” (PICO) guidelines. A total of 636 articles were found, and 14 articles were selected to be included in this review. The selected articles describe cases of full pulpotomies in mature permanent teeth with a diagnosis of irreversible pulpitis with a total of 34 (100%) successful cases, where 18 were men and 16 were women, with an average age of 19.20 ± 10.59 years and an average follow-up of 35.82 ± 26.39 months, with 12 months being the minimum follow-up time. The material used most frequently for obturation of the full pulpotomy was mineral trioxide aggregate in 16 cases (47.06%). Within the limitations of this review, full pulpotomy presents a high success rate regardless of the tooth, age, or sex as a treatment for teeth diagnosed with irreversible pulpitis.

## Introduction and background

According to the Global Burden of Disease study, oral conditions continue to be a substantial challenge in terms of population health. There were approximately 3.5 billion cases worldwide with oral conditions. Despite the efforts to eradicate dental caries, it remains highly prevalent and highly incident. It is estimated that 2.3 billion people had untreated caries in their permanent teeth in 2020 [1,2]. Dental caries is the most prevalent and salient of oral diseases worldwide. It is a multifactorial condition derived from demineralization of the hard tissues of the tooth. Most of the acids in caries are an outcome of bacteria metabolism, which is organized in highly structured bodies bound together by extracellular substances [3,4]. Because of the increasing dominance of a minimal intervention treatment philosophy in caries management and in pulpal treatments, the concepts of caries and its treatment have evolved over time, from the total removal of the tissue affected by caries to the selective and minimally invasive removal of the carious lesion [5].

Clinicians face teeth with advanced caries, close to the pulp chamber, where the biggest dilemma is how conservative treatment may be. Vital pulp therapy (VTP) is among the treatment technologies that exist in endodontics. VTP is the name given to all treatments whose objective is to preserve the vitality and maintain the integrity of the pulp to facilitate its repair after an injury resulting from trauma, caries, or restorative procedures. VTP procedures include indirect or direct pulp capping, and partial or full pulpotomy [6]. For many years, VTP was focused on the treatment of immature permanent teeth, with the main objective of supporting full root development. Today, the VTP approach has a broader outlook and has proven to be an effective alternative for the treatment of permanent teeth with irreversible pulpitis [7]. Currently, the American Association of Endodontists (AAE) diagnostic terminology classifies a vital pulp into one of three categories: "normal," "reversible pulpitis," or "irreversible pulpitis" (which may be symptomatic or asymptomatic). It defines irreversible pulpitis as a “clinical diagnosis based on subjective and objective findings indicating that the vital pulp is inflamed and incapable of healing” [8].

Until a few years ago, the treatment of choice for mature permanent teeth that had irreversible pulpitis was a root canal treatment or pulpectomy; the goals were to clean, shape, and seal the anatomical space where the pulp used to be. This treatment option is more involved and complex than the pulpotomy alternative. When a pulpectomy was performed on teeth diagnosed with irreversible pulpitis, 92% to 98% of cases were expected to be successful [9,10], compared to what has been reported for pulpotomies, where success was around 90% in permanent teeth [11,12]. This treatment option is less involved and complex than the pulpectomy alternative. It is important to mention that it was only very recently that a measure of standardization of clinical guidelines for the management of advanced caries and pulp exposure was attained, based on histological evidence [13].

There is sparse evidence about the success of pulpotomy as an effective treatment for permanent teeth with irreversible pulpitis. The objective of the present systematic review was to evaluate the current evidence about the full pulpotomy treatment of permanent teeth diagnosed with irreversible pulpitis.

## Review

Methods

Our rationale for selecting case reports for the present systematic review was that until 2021, the AAE made public the acceptance of performing pulpotomy on mature teeth with irreversible pulpitis. Previously, the only treatment for these teeth was pulpectomy. Due to the recentness of this new management for deep caries and pulp exposure, we believe it is important to report whether the treatment of mature permanent teeth with irreversible pulpitis is effective or not.

The screening process for the systematic review was carried out independently by two authors (LRB and VRI) following the Preferred Reporting Items for Systematic Reviews and Meta-analyses (PRISMA) guidelines [14]. Titles and abstracts of the articles identified with the search strategy were reviewed and contrasted against the inclusion criteria and each article was categorized as relevant, possibly relevant, and not relevant.

**Figure 1:**
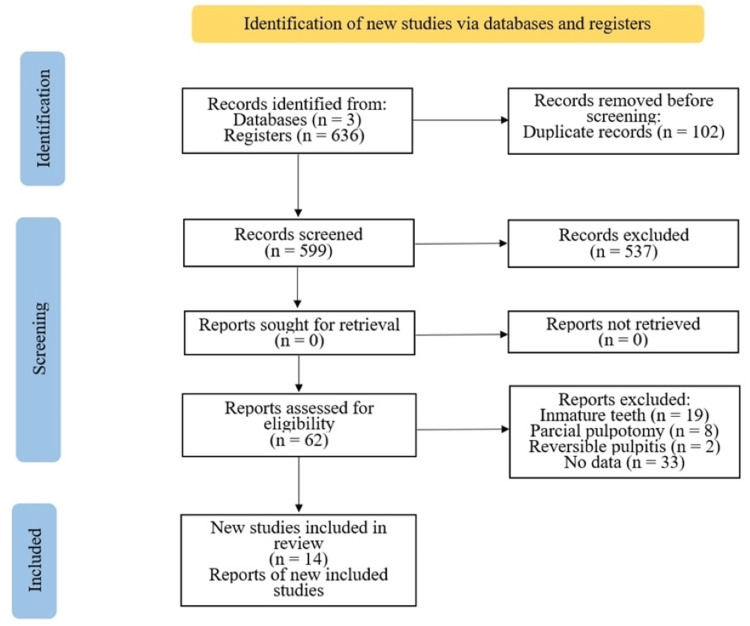
Flowchart of cases selection procedure and exclusion criteria

Rayyan software (Rayyan Systems Inc., Cambridge, MA) was used to manage the screening and review process, and then full-text copies of articles marked relevant and possibly relevant were obtained [15]. For the eligibility phase, two authors (LRB and VRI) categorized each article as include or exclude, and disagreements were resolved by a third author (CEMS). Figure 1 shows the selection process according to the PRISMA 2020 flow diagram [16].

Selection Criteria

An electronic search was performed to find articles in English where the treatment for irreversible pulpitis in permanent teeth was a full pulpotomy. Literature reviews, in vitro or animal studies, abstracts, and unpublished data were excluded. The intervention, control, and outcome parameters were selected following the “Population, Interventions, Control, and Outcome” (PICO) guidelines as follows: population: patients presenting irreversible pulpitis in a permanent tooth; intervention: full pulpotomy; control: other treatments for irreversible pulpitis (direct capping, pulpectomy treatment); outcome: clinical and radiographic success or efficacy of the treatment, follow-up time (minimum of three months, considering follow-ups of one month likely), and treatment eligibility.

Information Sources and Literature Search Strategy

An electronic search in English of published case reports was conducted using the PubMed, Google Scholar, and Scopus databases up to the year 2022. The search strategy was implemented in August 2022, with the following Medical Subject Heading (MeSH) terms used: “(case report OR case) AND pulpotomy AND (irreversible pulpitis OR inflamed pulp) AND (permanent dentition OR permanent teeth OR mature teeth OR permanent mature). All keywords were used in different combinations to exhaustively search for articles in the electronic databases.

Data Extraction and Analysis

Articles were selected for inclusion using the Rayyan platform for storage, duplicate identification, and article selection. The following process was followed: (1) initial search in the Google Scholar, PubMed, and Scopus databases. (2) Submit the results to the Rayyan platform. (3) Elimination of duplicates. (4) Two reviewers independently screened the results of the electronic search by title using the "blind" function, and when a title seemed relevant, the abstract was reviewed to determine its eligibility. (5) When the title and abstract were considered relevant, the full text of the article was reviewed. (6) The relevant data in the selected articles were identified and an Excel (Microsoft Corporation, Redmond, WA) database was created. When the two reviewers disagreed when choosing an article, a meeting was held to discuss and reach a consensus.

Quality Assessment

The quality of the selected articles was evaluated using the CARE Case Report guidelines [17]. A checklist was assembled to determine whether the selected articles offered the information necessary to be considered a case or a series of case reports.

Statistical Analysis

The statistical package Stata 16.0 (StataCorp LLC, College Station, TX) was used to carry out a descriptive analysis with frequencies and percentages and means and standard deviations reported.

Results

Findings From the Literature

A total of 636 articles were found that matched the terms and timeframe set for the bibliographic search: 526 were found in Scopus, 85 in Google Scholar, and 25 in PubMed. Of the 636 articles, 102 were eliminated due to being duplicate articles. After applying the inclusion criteria, 62 articles were evaluated to be chosen as shown in Figure 1.

A total of 14 articles were selected to be included in this review [18-31]. The selected articles reported cases or series of cases of full pulpotomies in mature permanent teeth with a diagnosis of irreversible pulpitis with a total of 34 cases (Table 1).

**Table 1:** Information on the articles included PAI: periapical index; CEM: calcium-enriched mix; MTA: mineral trioxide aggregate; PRF: platelet-rich fibrin.

Author	Year	Sex	Age	Tooth	Follow-up	Outcome	PAI	Material	Complications
Tran et al. [18]	2021	F	40	45	12	Effective	NO	Biodentine	NO
		F	25	36	24	Effective	NO	Biodentine	NO
Soni [19]	2016	M	12	46	18	Effective	NO	Biodentine	NO
Asgary et al. [20]	2017	M	36	26	24	Effective	NO	CEM	NO
		M	36	27	24	Effective	NO	CEM	NO
Asgary [21]	2011	M	15	46	24	Effective	NO	CEM	NO
Qudeimat et al. [22]	2016	F	10	46	67	Effective	NO	MTA	NO
		M	11	46	72	Effective	NO	MTA	NO
		M	11	36	72	Effective	NO	MTA	NO
		F	12	36	68	Effective	NO	MTA	NO
		M	11	46	19	Effective	NO	MTA	NO
		M	11	16	60	Effective	NO	MTA	NO
		M	11	36	67	Effective	NO	MTA	NO
		M	10	36	60	Effective	NO	MTA	NO
		M	10	26	60	Effective	NO	MTA	NO
		F	13	26	56	Effective	NO	MTA	NO
		F	14	37	55	Effective	NO	MTA	NO
		F	14	46	55	Effective	NO	MTA	NO
		M	12	26	37	Effective	NO	MTA	NO
Hiremath et al. [23]	2012	F	19	36	22	Effective	NO	PRF/MTA	NO
Mobarak et al. [24]	2021	F	28	46	12	Effective	NO	PRF/Biodentine	NO
		M	35	46	12	Effective	NO	PRF/Biodentine	NO
		M	40	46	12	Effective	NO	PRF/Biodentine	NO
Yousef et al. [25]	2021	M	39	46	18	Effective	NO	MTA	NO
Sharaan et al. [26]	2017	F	9	36	17	Effective	NO	CEM	NO
		F	14	46	17	Effective	NO	CEM	NO
		M	11	16	16	Effective	NO	CEM	NO
		F	15	36	16	Effective	NO	CEM	NO
		F	12	26	16	Effective	NO	CEM	NO
Munavalli et al. [27]	2018	F	19	36	18	Effective	NO	MTA	NO
Aleid [28]	2018	M	18	36	24	Effective	NO	PRF/MTA	NO
Dahiya et al. [29]	2020	M	21	46	12	Effective	NO	Biodentine	NO
Bhalla et al. [30]	2014	F	21	46	12	Effective	NO	MTA	NO
Asgary [31]	2022	F	39	37	120	Effective	NO	CEM	NO

Of the 34 cases, 18 were men and 16 were women, with an average age of 19.20 ± 10.59 years. No anterior teeth were found. The teeth that are most frequently diagnosed with irreversible pulpitis and receiving complete pulpotomy intervention were the right lower first molar and the left lower first molar (Table 2). The mean follow-up was 35.82 ± 26.39 months, with 12 months being the minimum follow-up time. The material used most frequently for full pulpotomy was mineral trioxide aggregate (MTA) in 16 cases (47.06%), and the second most used material was a “calcium-enriched mixture” (Table 3). All reported cases were effectively treated at the end of their follow-up time.

**Table 2:** Frequency and percentage of the tooth type variable

Tooth	Frequency	Percentage
16	2	6%
26	5	15%
27	1	3%
36	10	29%
37	2	6%
45	1	3%
46	13	38%
Total	34	100%

**Table 3:** Frequency and percentage of the variable type of material used CEM: calcium-enriched mix; MTA: mineral trioxide aggregate; PRF: platelet-rich fibrin.

Material	Frequency	Percentage
Biodentine	4	12%
PRF/Biodentine	3	9%
PRF/MTA	2	6%
CEM	9	26%
MTA	16	47%
Total	34	100%

Discussion

This systematic review of the literature was conducted to identify published clinical cases or series of cases where full pulpotomy treatment was performed on permanent teeth diagnosed with irreversible pulpitis. The intervention carried out in the 34 cases was effective, regardless of the material used, age, or sex. These results are like those found in a prior systematic review and meta-analysis where the clinical success of pulpotomy at 12 months was 97% and at 36 months was 94% [18]. In another systematic review reporting the treatment results of full and partial pulpotomies, a clinical success between 92% and 100% and 83%, respectively, at 12-month follow-up was found [32]. Based on the results of our and other research, it can be inferred that full pulpotomy is more successful than other pulp treatments such as direct pulp capping or partial pulpotomy. This difference could be explained by the histological conditions of the inflamed pulp tissue, the pulp diagnosis, and the lack of knowledge that previously existed when deciding about the appropriate treatment for pulp exposure. When a full pulpotomy is performed, the inflamed and injured tissue is most likely to be completely removed, unlike what happens in a partial pulpotomy [12,13].

It is important to mention that all the cases included in this review were classified as "successful" based on clinical signs and symptoms, as well as radiographic findings such as widening of the periodontal ligament space or the appearance of periapical radiolucent lesions that each author evaluated individually. Therefore, it is considered that to evaluate the therapeutic outcome in a more objective manner, a periapical index should be used, such as the index proposed by Orstavik et al. in 1986 [33] that allows evaluation of the radiographic periapical zone before, during, and at the end of the follow-up period, as used in other studies [34-37]. This should be complemented by clinical findings to make it possible to establish whether the case has been effective, uncertain, or ineffective, as proposed by Zanini et al. [38].

Various materials have been used over time as pulp caps. Calcium hydroxide is one of the first materials used that has beneficial characteristics, one of which is the stimulation of mineralized tissue [39]. Some new materials have emerged, such as “endodontic bioactive cement” and “calcium silicate cement” [40,41]. These materials largely share a common feature, bioactivity, which means that they release calcium ions, produce calcium hydroxide, form a layer between the cementum and the dentin wall of apatite crystals, and have an excellent seal [42]. There is substantial evidence in the literature supporting the success of such materials in pulpotomies in permanent teeth [22,43,44]. However, there has not been an agreement on which material is the best.

Strengths and limitations

To the best of our knowledge, this systematic review is the first to examine current evidence from case reports where the treatment for permanent teeth diagnosed with irreversible pulpitis was a full pulpotomy. However, the limitations of the present study were the lack of a statistical analysis that allows more solid conclusions, given the limited number of reports on the subject. On the other hand, we know that there could be some bias since success stories are generally published, while clinical failures may be less attractive to journals.

## Conclusions

Full pulpotomy is a highly successful treatment for teeth with advanced caries, offering the opportunity to avert a full pulpectomy.

The high success rate for full pulpotomy treatments, regardless of tooth type, age, or sex, suggests that this procedure is less invasive and aims to save the pulp tissue in teeth diagnosed with irreversible pulpitis. However, more studies comparing outcomes directly between pulpectomy and pulpotomy treatments in the same patient or across matched categories of teeth, with clearly defined inclusion/exclusion criteria, are needed to quantify their effectiveness.
